# Recent Advances in the High-Value Conversion of Alkenes Induced by Electrochemistry

**DOI:** 10.3390/molecules31061027

**Published:** 2026-03-19

**Authors:** Xing’an Liang, Haolin Wang, Wei Xie, Zhenhua Liu, Dongmiao Qin

**Affiliations:** School of Environmental and Food Engineering, Liuzhou Polytechnic University, Liuzhou 545006, China; wanghaolin@lzpu.edu.cn (H.W.); ivy121@163.com (W.X.); oaklzh@163.com (Z.L.); qindongmiao@lzpu.edu.cn (D.Q.)

**Keywords:** electrochemistry, alkene, high-value conversion

## Abstract

Over the past few decades, electrosynthesis has advanced significantly, enabling numerous valuable transformations for synthetic chemists. Olefins are inexpensive, readily available industrial feedstocks extensively used in organic synthesis. Therefore, achieving high-value transformation of olefins is of great value. However, the use of stoichiometric oxidants and the generation of stoichiometric waste hinder its broader application. Utilizing electrochemistry to achieve high-value transformations of olefins represents a green, environmentally friendly, and sustainable strategy, since it eliminates the need for external oxidants. This review discusses recent advances in the high-value conversion of alkenes induced by electrochemistry. The article introduces two modes of electrochemical olefin transformation, discussing both synthetic applications and mechanistic studies. It highlights their advantages and suggests future directions to tackle the existing challenges in this synthetic domain.

## 1. Introduction

Electricity is a clean energy source which is widely used in daily life. In the 18th century, after the discovery of the acrylonitrile polymerization and the carboxylic acids polymerization by electrochemistry, the application of electrochemistry in organic synthesis attracted extensive attention from chemists [1,2,3]. Electrosynthesis is regarded as one of the most environmentally friendly way to organic synthesis. Because it uses electrons as a mass-free reagent to replace stoichiometric chemicals, enabling functionalization processes and eliminating wast. Furthermore, electron transfer reverses the polarity of functional groups, creating unique possibilities unattainable through conventional chemical methods [4,5,6,7,8].

### 1.1. Electrosynthesis: Oxidation and Reduction Electrosynthesis

**Figure 1:**
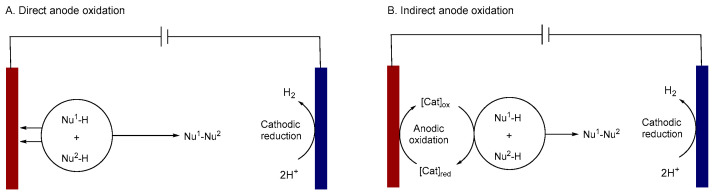
(**A**). Direct anode oxidation; (**B**). Indirect anode oxidation.

In electrosynthesis, electron transfer at an electrode drives the desired transformation by initiating a subsequent chemical reaction in a molecule (substrate or mediator) [9,10]. The reaction can be performed via oxidation or cathodic reduction electrolysis. Electrochemical oxidation can be divided into two mechanisms: direct oxidation and indirect oxidation. Direct oxidation operates through a two-step process (Figure 1A). First, a heterogeneous electron transfer occurs directly between the electrode and the target substrate, creating a reactive intermediate. Subsequently, this intermediate undergoes a chemical reaction with another molecule or functional group to form the final product. Rapid reaction rate and high efficiency are the advantages of direct oxidation. However, in this mode, the progression of substrate oxidation is difficult to control, which frequently leads to undesired side reactions [11,12].

The indirect oxidation involves a redox mediator that acts as an electron shuttle (Figure 1B). Because its redox potential is lower than the substrate, the mediator is preferentially oxidized at the electrode to produce a reactive species, which subsequently carries out the desired transformation of the substrate. Indirect oxidation can overcome the inherent limitations of direct oxidation: (a) avoidance of issues associated with heterogeneous electron transfer, such as high overpotentials; (b) operation at applied potentials lower than the substrate’s redox potential; (c) acceleration of the reaction kinetics; and (d) achievement of higher selectivity by bypassing competing side reactions. Common mediators include organic small molecules and transition metals. Over the past two decades, significant advances have been made in the use of transition-metal molecular electrocatalysts, which have realized a wide range of fascinating chemical transformations [6,13].

### 1.2. High-Value Conversion of Alkenes

**Figure 2:**
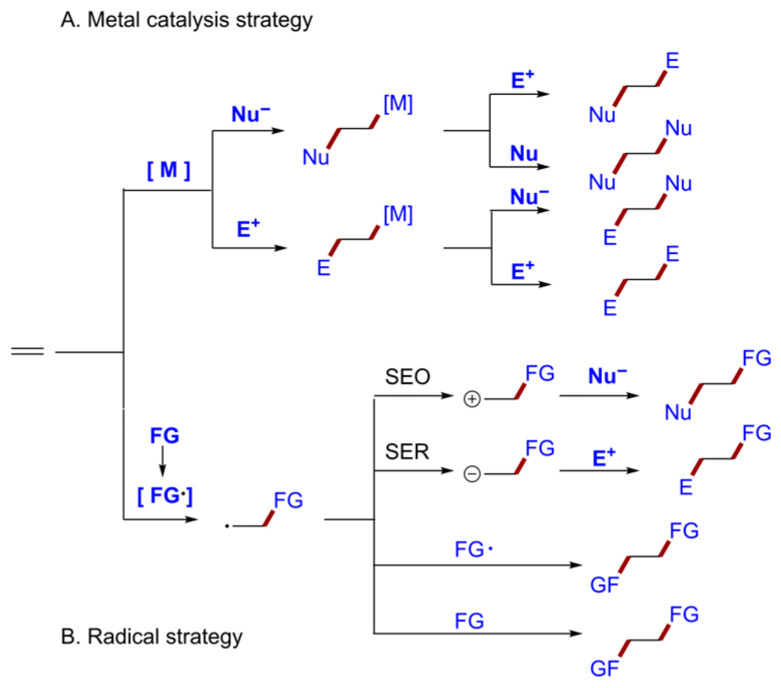
Brief Summary of the Alkene Difunctionalization.

As inexpensive and readily available chemical feedstocks, alkenes are widely used in organic synthesis. The unique chemical structure of alkenes provides an opportunity for multicomponent cross-coupling reactions. Hence, constructing medicinally or functionally important compounds from these olefins has driven the pursuit of efficient synthetic methods. Prominent among these is alkene difunctionalization, a highly efficient strategy for building molecular complexity through the simultaneous addition of two functional groups (Figure 2) [7,14,15,16]. Conventionally, alkene difunctionalization has primarily relied on transition-metal-catalyzed cross-coupling with organohalides and/or organometallics (Figure 2A). Despite its utility, this approach faces limitations such as the need for expensive metal catalysts, often requiring pre-functionalized starting materials, and sometimes harsh reaction conditions. In recent years, radical-mediated difunctionalization strategies have emerged as a powerful alternative. Utilizing either an oxidation strategy or transition-metal redox catalysis, these methods have significantly broadened the substrate scope, enabling the direct functionalization of even saturated hydrocarbons (Figure 2B). However, these approaches inherently require stoichiometric oxidants or additives and often demand expensive metal/ligand catalysts, typically with heating. Therefore, developing an efficient and green method for the functionalization of olefins is highly valuable.

Electrochemical catalysis has emerged as a powerful and sustainable platform for innovating synthetic reactions that build molecular complexity, finding broad application in both academia and industry [17,18]. With the progress in electrochemical synthesis, utilizing electrochemistry to enable the value-added transformation of alkenes has been attracting widespread attention among chemists. Electrocatalytic functionalization serves as a novel platform for reaction development, providing benefits that include cleaner conditions and expanded reactivity [19,20,21]. This approach provides key benefits over conventional methods. First, selectivity is precisely tunable through electrochemical parameters (potential, current, electrode material), affording high chemo-, regio-, and stereo-control with minimal byproduct formation. Second, electricity serves as a clean energy source, diminishing or eliminating the need for chemical oxidants/reductants and precious metal catalysts, which streamlines the reaction and makes it more sustainable. Finally, electrochemical transformations can achieve high atom- and step-economy, leading to efficient material use and reduced waste.

**Figure 3:**
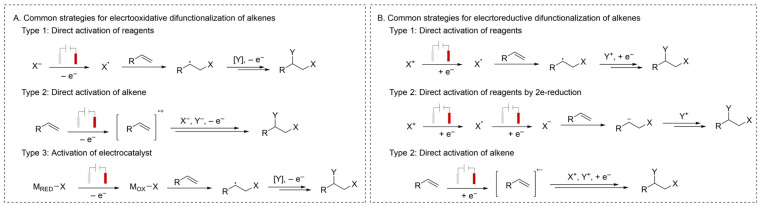
(**A**). Common strategies for electrooxidative difunctionalization of alkenes; (**B**). Common strategies for electroreductive difunctionalization of alkenes.

It is reported that the oxidation potential of alkenes is approximately 2.0 V [22]. The electrochemical approaches for their oxidation functionalization can be primarily divided into three pathways (Figure 3A). (1) generating a radical intermediate via direct activation of a nucleophile to initiate alkene addition; (2) forming a radical cation through direct activation of an alkene prior to difunctionalization; and (3) employing a redox catalyst that governs the addition of radicals across an alkene [23,24,25].

In contrast, electrochemical reductive functionalization of alkenes also represents one of the most efficient methods for their value-added transformation. From a mechanistic perspective, these recent advances are broadly classified into three categories (Figure 3B): (1) generating a radical intermediate via direct activation of an electrophile to initiate alkene addition; (2) generating an anionic intermediate via direct 2e-reduction in an electrophile to initiate alkene addition and (3) forming a radical anion through direct activation of an alkene prior to difunctionalization [26,27,28]. A common feature of oxidation strategies is their reliance on oxidative activation. In contrast, the development of electroreductive approaches for alkene difunctionalization remains comparatively underdeveloped. Over the years, a variety of reports have been published that summarize the impressive advances made in the field of electrochemistry alkene functionalization [8,24,29]. This review summarizes recent advances in the electrochemical functionalization of alkenes. This review aims to promote the adoption of organic electrosynthesis, a technique with considerable potential, into the standard synthetic organic toolbox.

## 2. Functionalization of Alkenes

### 2.1. Anodic Alkene Functionalization

#### 2.1.1. C–C Bonds Formation

**Figure 4:**
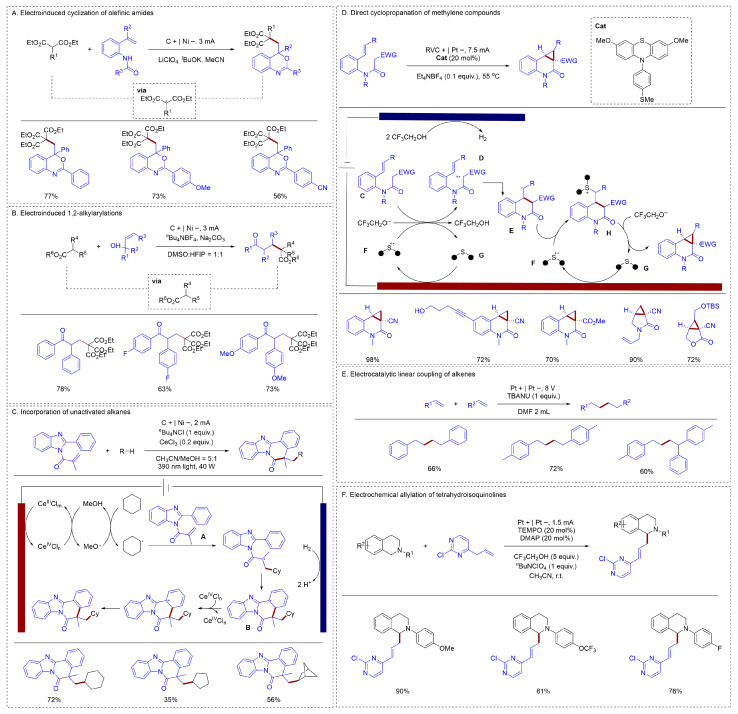
(**A**). Electroinduced cyclization of olefinic amides; (**B**). Electroinduced 1,2-alkylarylations; (**C**). Incorporation of unactivated alkanes; (**D**). Direct cyclopropanation of methylene compounds; (**E**). Electrocatalytic linear coupling of alkenes; (**F**). Electrocatalytic linear coupling of alkenes.

Anodic oxidation, as the most common mode of electrochemical synthesis, has been successively reported by chemists in recent years. The formation of carbon–carbon bonds via electro-oxidation offers a direct and efficient route to synthesizing various compounds. A direct electro-oxidation strategy was developed by Zeng to generate these electrophilic carbon radicals without the need for a catalyst to achieve the functionalization of alkenes [30] (Figure 4A). Using triethyl methanetricarboxylate as a carbon radical precursor, he generated carbon radicals via a direct oxidation strategy. These radicals were subsequently trapped by alkenes to achieve alkene functionalization. He also achieved the functionalization of enols using a similar strategy [31] (Figure 4B). Although this method can efficiently construct C–C bonds of alkenes, its substrate scope was severely limited. It only enabled the generation of carbon radicals via the activation of activated C–H bonds, while the activation of inert alkanes remained a significant challenge.

To achieve the activation of simple alkanes, Zeng reported a novel cerium-catalyzed electrophotoredox strategy for the radical cascade synthesis of alkylated benzimidazo-fused isoquinolinones and related *N*-heterocycles from unactivated alkanes [32] (Figure 4C). A key feature of this electrophotocatalytic strategy was its high step- and atom-economy. Based on preliminary mechanistic studies, it was proposed that MeOH promoted the formation of an active Ce(IV) species, which was responsible for generating alkyl radicals and thus initiating the cascade. The proposed mechanism for the tandem reaction began with the oxidation of Ce^III^, yielding the MeO-Ce^IV^Cl_n−1_ complex. Subsequently, this complex underwent homolytic cleavage through a ligand-to-metal charge transfer (LMCT) process under light irradiation, generating a methoxy radical. Subsequently, the cyclohexyl radical, formed via hydrogen atom transfer (HAT) from cyclohexane, underwent a radical addition/cyclization cascade with **A** to give intermediate **B**; Final SET oxidation by Ce(IV) and deprotonation then afforded the product. He achieved the activation of simple alkanes by utilizing an indirect activation approach combined with photocatalysis. A variety of simple alkanes, such as cyclohexane and cyclopentane, could all be obtained with moderate yields to give the target compound.

Also employing indirect oxidation, a method was presented by Xu for the electrocatalytic cyclopropanation of active methylene compounds using an organic catalyst [33] (Figure 4D). Mechanistic studies indicated that the transformations occur via a radical–polar crossover mechanism, leading to the simultaneous formation of two carbon–carbon bonds within the newly generated cyclopropane ring. The catalytic cycle was initiated by the one-electron oxidation of **G** at the anode, producing the stable radical cation **F**. This oxidized catalyst then mediated the oxidation of substrate **C**, a step potentiated by the concurrent cathodic generation of CF_3_CH_2_O^−^ base. The reaction proceeded via deprotonation/electron transfer to deliver electrophilic radical **D** and regenerated **G**. Intramolecular cyclization of **D** afforded radical **E**, which was finally intercepted by **F** in a radical-radical coupling step, yielding sulfonium **H**. Finally, a nucleophilic substitution by CF_3_CH_2_O^−^ on sulfonium **H** furnished the cyclopropanation product and liberated the catalyst **G**.

Gong utilized the mode of forming olefin radical cations via oxidized olefins to achieve the dimerization reaction of olefins [34] (Figure 4E). This approach enabled both homo- and cross-coupling of alkenes and has been effectively utilized in the synthesis of bioactive compounds. Electroredox catalysis offered a direct and efficient pathway to generate alkene-derived radical anions, thereby facilitating the broad use of these highly reactive intermediates in chemical synthesis under mild, metal-free, and oxidant/reductant-free conditions. Various aryl alkenes could achieve coupling reactions with high yields and selectivity, but the coupling reaction of alkyl alkenes remained a challenge. Mei employed a similar strategy to achieve an efficient α-allylation of THIQs under mild conditions, offering a wide tolerance for various functional groups in the products [35] (Figure 4F). The strategy was easily scalable, significantly enhancing the efficiency and practicality of the tetrahydroisoquinoline allylation.

**Figure 5:**
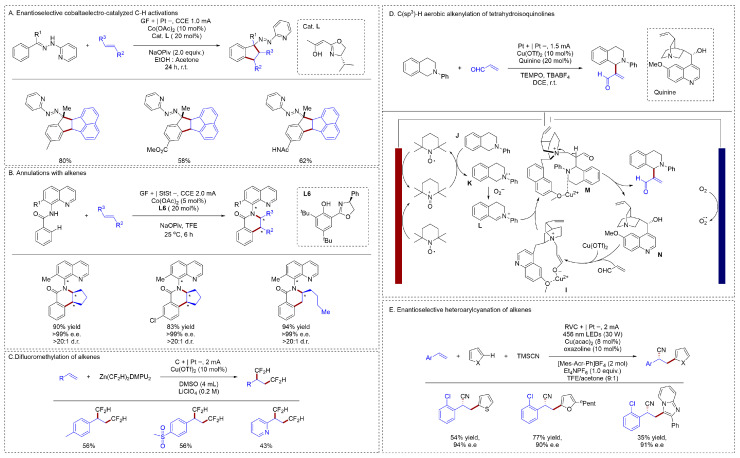
(**A**). Enantioselective cobaltaelectro-catalyzed C–H activations; (**B**). Annulations with alkenes; (**C**). Difluoromethylation of alkenes; (**D**). C(sp3)-H aerobic alkenylation of tetrahydroisoquinolines; (**E**). Enantioselective heteroarylcyanation of alkenes.

The combination of electrochemistry and metal catalysis enables more complex chemical transformations. Ackermann had developed a mild, operationally simple, and scalable method for the enantioselective cobaltaelectrocatalyzed annulation of aryl hydrazones with olefins [36] (Figure 5A). This reaction provided direct access to chiral Fsp^3^-rich molecules and was distinguished by its ability to forge C(sp^3^)−C(sp^3^) bonds with high enantio- and diastereoselectivity. A key innovation was the implementation of a novel class of chiral κ2-*N*,*O*-oxazoline preligands, whose modular stereoelectronic tunability offered significant potential for broader applications in asymmetric transition metal catalysis. Various electron-deficient and electron-rich aryl alkenes could yield the target compounds in high yields; However, the activation functionalization of alkyl alkenes remained to be solved. An electrochemical cobalt-catalyzed system enabled the one-step construction of a chiral carbon-nitrogen axis alongside up to four contiguous stereocenters, which had also been reported [36] (Figure 5B). This electrocatalytic method exhibited good functional group tolerance and provided a resource-efficient route to diverse chiral isoquinolones with excellent selectivity. Employing this strategy enabled the asymmetric functionalization of alkyl alkenes, and various cycloalkanes and chain alkenes could afford the target compounds with high yields and selectivity. Compared with cobalt, the less expensive metal copper has also been applied to the electro-mediated construction of C–C bonds in alkenes. Kin presented a copper-electrocatalyzed approach for the vicinal bis-difluoromethylation of alkenes [37] (Figure 5C). Conventionally used as an anionic transmetalating reagent, Zn(CF_2_H)_2_(DMPU)_2_ was electrochemically oxidized in this system to serve as a CF_2_H radical source, allowing for the introduction of the first CF_2_H group at the terminal position of alkenes. Mechanistic investigations indicated that a copper catalyst intercepted the resulting secondary radical, and the reaction proceeded via a Cu(III) intermediate to facilitate reductive elimination. This step enabled the installation of the second CF_2_H group at the internal position. Both arenes and heteroarenes were well tolerated in this system, affording the products in moderate yields.

Besides difunctionalization, electrochemically induced C–H functionalization of olefins was also reported recently. Mei had developed an efficient strategy for the alkenylation of tetrahydroisoquinolines [38] (Figure 5D). Their approach employed TEMPO as a mediator and quinine as a catalyst, enabling an electrochemical enantioselective alkenylation. The reaction proceeded through the coupling of electrochemical and catalytic cycles. Anodic oxidation generated TEMPO^+^, which oxidized substrate **J** to **K**. This intermediate was then oxidized by cathodically generated superoxide (O_2_•^−^) to form the key iminium species **L**. Concurrently, in the separate catalytic cycle, a copper salt activated acrolein towards nucleophilic attack by **N**, producing intermediate **I**. The copper center then orchestrated the coupling by coordinating both **I** and **L** to assemble the transition state **M**. Product was formed upon deprotonation of **M**, with regeneration of the catalyst. Xu described a photoelectrocatalytic approach for the enantioselective heteroarylcyanation of aryl alkenes using unfunctionalized heteroarenes via C–H functionalization [39] (Figure 5E). This photoelectrochemical asymmetric catalysis (PEAC) strategy merged photoredox catalysis with asymmetric electrocatalysis, enabling the construction of two C–C bonds through a hydrogen evolution pathway and eliminating the requirement for exogenous chemical oxidants.

Overall, electrochemistry serves as an effective approach for achieving C–C bond construction in alkenes. Direct oxidation enables the efficient, convenient, and rapid formation of C–C bonds for alkenes that are readily oxidized. However, for substrates with high oxidation potentials, direct oxidation methods struggle to achieve activation. Moreover, this strategy faces challenges in regulating site selectivity and diastereoselectivity. In contrast, indirect oxidation strategies, through the introduction of metal or small-molecule mediators, effectively address the activation challenges associated with substrates with high oxidation potentials. The incorporation of metal mediators also facilitates the regulation of substrate selectivity. Nevertheless, indirect oxidation approaches often involve complex reaction systems and suffer from lower efficiency. These two strategies complement each other, leveraging their respective strengths to overcome challenges in constructing C–C bonds across various alkene substrates.

#### 2.1.2. C–N Bonds Formation

**Figure 6:**
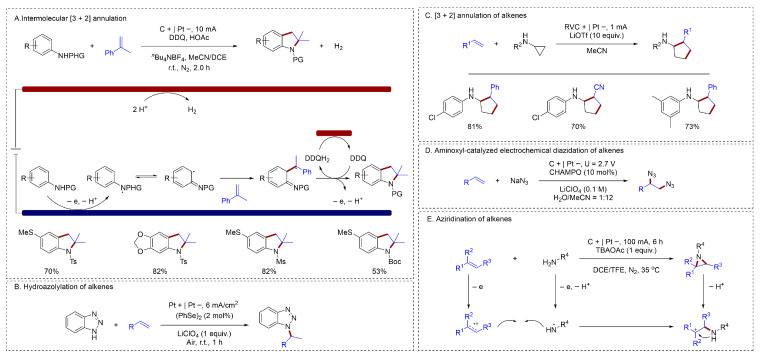
(**A**). Intermolecular [3 + 2] annulation; (**B**). Hydroazolylation of alkenes; (**C**). [3 + 2] annulation of alkenes; (**D**). Aminoxyl-catalyzed electrochemical diazidation of alkenes; (**E**). Aziridination of alkenes.

Anodic oxidation provides access to reactive radical cations or radicals, which serve as valuable intermediates for constructing biologically important N-heterocycles. Lei established an innovative electrochemical strategy that enabled the intermolecular [3 + 2] annulation of anilines and alkenes [40] (Figure 6A). This approach eliminated the need for external oxidants, providing a straightforward and atom-economical pathway to synthesize functionalized indolines. On the basis of mechanistic studies, the authors proposed that the oxidation and deprotonation of amine compounds to generate nitrogen-centered radical intermediates was key to this reaction. The nitrogen-centered radical was then captured by the alkene, followed by further oxidation and cyclization to afford the target compound. Meanwhile, hydrogen was formed as the only byproduct. Various types of 2-Phenyl-1-propenes were well tolerated in this system; However, the construction of C–N bonds with simple aryl alkenes and even alkyl alkenes remained challenging. A new electroselenocatalytic method had been established by Zeng for achieving the intermolecular hydroazolylation of electron-rich alkenes with azoles [41] (Figure 6B). This electroselenocatalytic protocol offered significant advantages, including low catalyst loading, external oxidant-free conditions, high current efficiency, and excellent regioselectivity. Its broad functional group tolerance was further demonstrated by the successful azolylation of natural product-derived alkenes.

Chen reported a novel and notable electrochemical redox-neutral reaction [42] (Figure 6C). A catalyst-free [3 + 2] annulation between N-cyclopropylamines and alkenes was achieved through direct electrolysis. Mechanistic studies, including the use of a divided cell, catalytic current measurements, and online MS monitoring of the key electron transfer step between the product radical cation and the reactant, supported a chain reaction mechanism. These methods could only achieve the activation of activated secondary amines to generate nitrogen-centered radicals, while the activation of unactivated primary amines remained a challenge. A new aminoxyl radical catalyst, CHAMPO, had been developed by Lin for the electrochemical diazidation of alkenes [43] (Figure 6D). Indirect oxidation overcame the difficulty in directly oxidizing azide anions, generating azidyl radicals to achieve alkene diazidation. Aziridines derived from bioactive molecules could also be constructed at the anode under electrochemical conditions through an alkene radical cation process [44] (Figure 6E). The initial stage involved a dual oxidation at the anode: on the one hand, sulfonamide was oxidized and lost a proton to furnish an *N*-centered radical; Meanwhile, alkene was oxidized to form the corresponding alkene radical cation. Then, a radical/radical cation cross-coupling occurred. Finally, further oxidation followed by cyclization yielded the target product.

**Figure 7:**
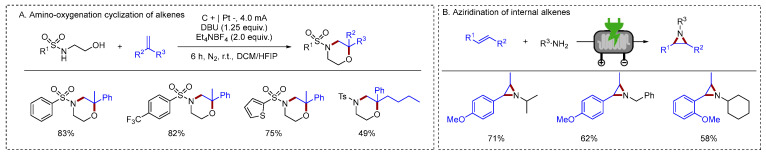
(**A**). Amino-oxygenation cyclization of alkenes; (**B**). Aziridination of internal alkenes.

In addition to C–N bond formation via nitrogen radicals generated from N–H activation, another approach involves the generation of olefin radical cations through alkene activation. Electrochemical amino-oxygenation cyclization of alkene radical cations with bisnucleophiles was also reported by Lei [45] (Figure 7A). Their method facilitated the in situ generation of alkene radical cations, enabling the efficient and selective synthesis of six- to eight-membered *N/O*-heterocycles from simple precursors. It provided streamlined access to complex molecular architectures crucial for medicinal chemistry and materials science. Noël had made significant contributions to the functionalization of olefins using flow electrochemistry. He described an electrochemical strategy for the aziridination of internal alkenes using primary amines [46] (Figure 7B). This continuous-flow strategy achieved reaction times as short as 5 min, along with high yields and broad applicability. Simple aryl alkenes enabled the construction of aziridines in high yields.

**Figure 8:**
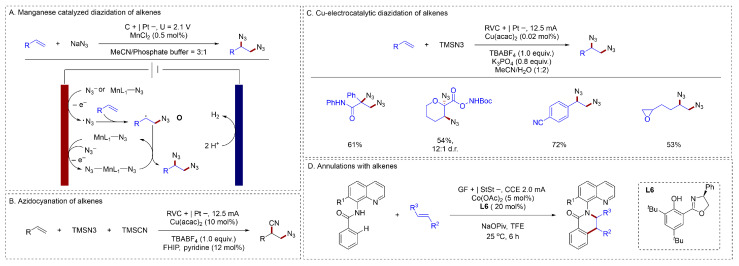
(**A**). Manganese-catalyzed diazidation of alkenes; (**B**). Azidocyanation of alkenes; (**C**). Cu-electrocatalytic diazidation of alkenes; (**D**). Annulations with alkenes.

Organic azides serve as versatile building blocks with broad utility across multiple disciplines, including chemical biology, materials science, and organic synthesis. Compared to the indirect oxidation approach for alkene diazidation [43], the use of a metal catalyst significantly reduces the catalyst loading. Lin described manganese porphyrin-based electrocatalysts employing sodium azide for the vicinal diazidation of alkenes [47] (Figure 8A). Mechanistic studies demonstrated that second-sphere hydrogen-bond donors played a crucial role in facilitating the stabilization of key reaction intermediates. The reaction began with the formation of [Mn(L_1_)N_3_] from [Mn(L_1_)Cl] and N_3_^−^. Subsequent oxidation of this complex achieved [Mn(L_1_)(N_3_)_2_], which fragmented to generate a free azidyl radical. This radical added to alkene, forming the transient radical intermediate **O**. Given that the direct cross-coupling of two such transient radicals was statistically unfavorable, the second C–N bond formation instead proceeds via an azidyl transfer from the anodically generated [Mn(L_1_)(N_3_)_2_] to **C**, ultimately delivering the diazide product. Xu described an unprecedented electrochemical strategy for alkene azidocyanation that accommodates both alkyl and aryl alkenes [48] (Figure 8B). Xu had also reported numerous studies in the area of indirect oxidation. He reported a scalable copper-electrocatalytic method for alkene diazidation [49] (Figure 8C). This reaction operated with only 0.02 mol % (200 ppm) of copper(II) acetylacetonate as the precatalyst and required no exogenous ligands. Both aryl and alkyl alkenes underwent diazidation with high yields. An electrochemical cobalt-catalyzed system enabled the one-step construction of a chiral carbon-nitrogen axis alongside up to four contiguous stereocenters had also been reported [50] (Figure 8D). This electrocatalytic method exhibited good functional group tolerance and provided a resource-efficient route to diverse chiral isoquinolones with excellent selectivity.

**Figure 9:**
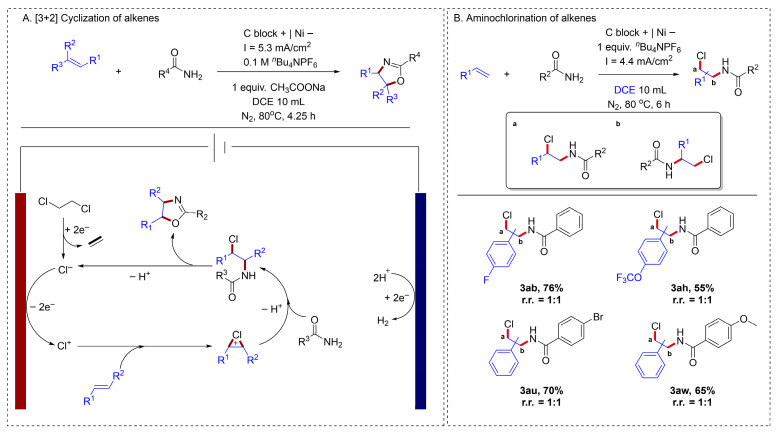
(**A**). [3 + 2] Cyclization of alkenes; (**B**). Aminochlorination of alkenes.

For olefins with higher oxidation potentials, it is difficult to activate them through either direct or indirect oxidation approaches. Developing new methods to oxidize olefins with high oxidation potentials is imperative. In 2021, Lei reported an interesting approach for the electrochemical activation of alkenes [51] (Figure 9A). Mechanistic studies revealed a key sequence: first, chloride anions generated from inexpensive 1,2-dichloroethane via cathodic reduction were oxidized at the anode. These activated chlorine species then reacted with alkenes to form chloronium intermediates, which were essential for the ensuing cyclization. Finally, chloronium intermediates were trapped by the amide, followed by cyclization to afford the target product. This method of generating chloronium ions via the electrochemical reduction in DCE offered a new strategy for the electrochemical activation of alkenes. Liang also described an electrochemical method that enabled the amidochlorination of low-cost styrenes with amides, without requiring additional chlorinating reagents [52] (Figure 9B). Despite its limited site selectivity, this reaction could be controlled to stop specifically at the chloroamination step, preventing further cyclization to oxazoline products. Simple aryl alkenes were well tolerated in this system; however, the activation of simple alkyl alkenes remained a challenge.

Electrochemistry represents an efficient strategy for constructing C–N bonds in alkenes. Anodic oxidation effectively activates stable N–H compounds, enabling the formation of C–N bonds in alkenes. However, for certain nitrogen-containing compounds that are prone to self-coupling and instability, indirect oxidation methods are required to achieve C–N bond formation. Indirect oxidation also facilitates the asymmetric construction of C–N bonds in alkenes, addressing the challenge of selectivity regulation.

#### 2.1.3. C–O Bonds Formation

**Figure 10:**
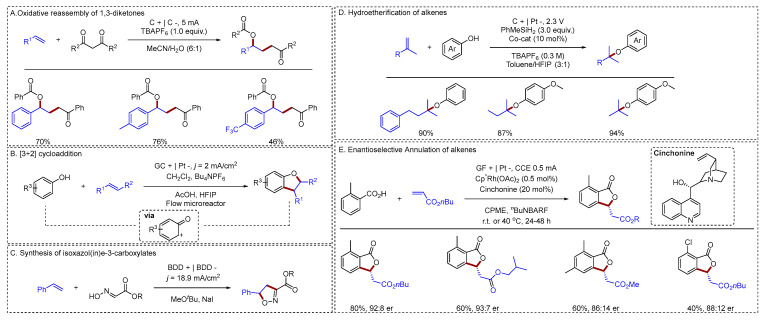
(**A**). Oxidative reassembly of 1,3-diketones; (**B**). [3 + 2] cycloaddition; (**C**). Synthesis of isoxazol(in)e-3-carboxylates; (**D**). Hydroetherification of alkenes; (**E**). Enantioselective Annulation of alkenes.

Anodic oxidative C–O bond coupling provides a key strategy for synthesizing oxygen-containing compounds. Ren reported an electrochemical strategy that cleaved and reassembled 1,3-diketones with aryl alkenes and water, enabling the direct synthesis of diverse 1,4-ketoalcohol derivatives in good to high yields [53] (Figure 10A). This work represented the first formal C–C bond cleavage of 1,3-diketones coupled with alkene insertion via electro-oxidation. Various aryl alkenes could afford the target compounds in high yields; However, alkyl alkenes were not compatible with this system. Mahito Atobe developed an electrochemical [3 + 2] cycloaddition of phenols and alkenes within a laminar-flow microreactor [54] (Figure 10B). Utilizing a flow microreactor enabled the electrochemical production of dihydrobenzofurans with minimal waste and high energy efficiency, accomplished at significantly reduced electrolyte concentrations and cell voltage. An electrochemical synthesis of isoxazol(in)e-3-carboxylates had been reported by Waldvogel [55] (Figure 10C). This protocol proved to be broadly versatile, as evidenced by >30 diverse and highly functionalized examples. A successful demonstration of its sustainability was the synthesis of an isoxadifen-ethyl derivative, which circumvented hazardous reagents and reduced waste generation. Kim reported a general electrocatalytic method for the modular synthesis of alkyl aryl ethers from diverse alkenes and phenols [56] (Figure 10D). This hydroetherification proceeds via an electrochemically generated cobalt-hydride catalyst, which mediates a radical-polar crossover of the alkene. This key step generated a cationic intermediate that was efficiently captured by even challenging phenolic nucleophiles. Employing this strategy, alkyl alkenes enabled the construction of C–O bonds with high yields. The Cyclofunctionalization reactions of olefins had also been reported. A novel electrochemical domino catalysis strategy for the synthesis of chiral phthalides was presented by Ackermann [57] (Figure 10E). In contrast to traditional methods, this approach employed an achiral Cp*-rhodium catalyst and a readily available chiral Brønsted base to drive an enantioselective C–H activation/annulation between benzoic acids and alkenes. This offered a streamlined, environmentally friendly alternative, leveraging electric current as a sustainable oxidant to assemble the desired products in good enantioselectivity.

#### 2.1.4. C–S Bonds Formation

**Figure 11:**
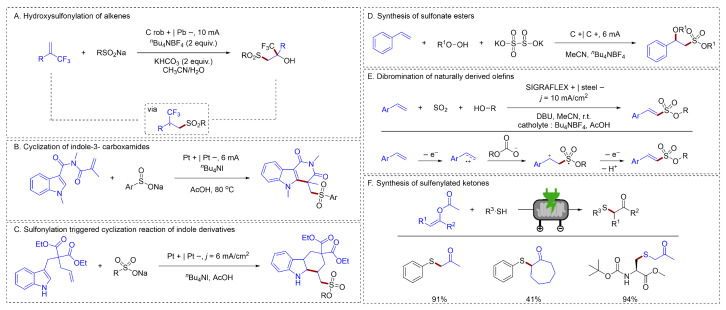
(**A**). Hydroxysulfonylation of alkenes; (**B**). Cyclization of indole-3- carboxamides; (**C**). Sulfonylation triggered cyclization reaction of indole derivatives; (**D**). Synthesis of sulfonate esters; (**E**). Dibromination of naturally derived olefins; (**F**). Synthesis of sulfenylated ketones.

Alkenesulfonates have recently been found to exhibit unique biological activity. Lei had developed a direct electrochemical method for the hydroxysulfonylation of α-CF_3_ alkenes, producing α-trifluoromethyl tertiary β-hydroxysulfones in up to 88% yield [58] (Figure 11A). This catalyst-free protocol exhibited broad compatibility with various sodium sulfinates. Crucially, the process avoided the reduction in the alkene and competing E1cB-type fluoride elimination at the cathode, with hydrogen gas as the sole byproduct. Han had established multiple methods for the cyclization-functionalization of indoles with alkenes. An efficient electrochemical radical cyclization between N-acryloylindole-3-carboxamides and sodium sulfinates had been established by Han [59] (Figure 11B), providing direct access to multi-substituted γ-carbolinones with yields as high as 70%. In addition, Han also developed an efficient electrochemical method to synthesize functionalized tetrahydrocarbazoles from readily available indole derivatives and sodium sulfinates [60] (Figure 11C). This transformation proceeded via a sequence of sulfonylation, cycloaddition, and deprotonation, and tolerated a wide range of electronically and sterically diverse substituents on both coupling partners. Sulfonate esters were valuable scaffolds found in both drugs and natural products. They all chose sodium sulfinate as the sulfur source. Although the efficiency is high, there are certain limitations in terms of substrate applicability. The construction of C–S bonds in alkenes via S-H species remained to be solved.

To achieve the construction of more types of C–S bonds, Han had developed an electrochemical method that accomplished the difunctionalization of alkenes to *β*-alkoxyl sulfonate esters under transition-metal-free conditions, through sequential C–S, S–O, and C–O bond formations [61] (Figure 11D). A novel electrochemical multicomponent reaction was also developed by Waldvogel for the synthesis of alkyl alkenesulfonates [62] (Figure 11E). This method utilized readily available starting materials—styrenes, SO_2_ solution, and alcohols—alongside cost-effective electrodes and separators. Its broad applicability was demonstrated through a substrate scope of 44 examples, achieving yields of up to 81% while exhibiting excellent regio- and stereoselectivity. Noël investigated the electrochemical sulfenylation between thiophenols/thiols and enol acetates to yield α-sulfenylated ketones, both in batch and continuous flow modes [63] (Figure 11F). Employing flow electrochemical methods could substantially enhance reaction productivity, thus facilitating industrialization. However, the high cost of flow electrochemical devices and the thermal effects associated with large cells were still significant obstacles to the industrialization of flow electrochemistry.

#### 2.1.5. C–X Bonds Formation

**Figure 12:**
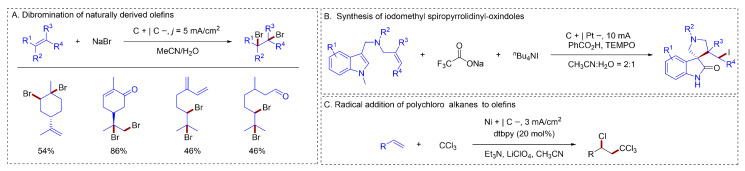
(**A**). Dibromination of naturally derived olefins; (**B**). Synthesis of iodomethyl spiropyrrolidinyl-oxindoles; (**C**). Radical addition of polychloro alkanes to olefins.

Alkyl halides are important synthetic intermediates in organic synthesis. The construction of C–X bonds through the functionalization of alkenes represents a highly efficient approach. Waldvogel developed a simple electrochemical method for the selective dibromination of naturally occurring alkenes [64] (Figure 12A). This approach elegantly avoided hazardous Br_2_ or its analogs by employing inexpensive and benign sodium bromide, which acted as both the bromine source and the supporting electrolyte, in combination with sustainable carbon-based electrodes. Han established a novel electrochemical multicomponent cascade strategy using indole-tethered alkenes, CF_3_SO_2_Na, and *n*-Bu_4_NI, enabling the rapid construction of spiropyrrolidinyl-oxindoles in good yields [65] (Figure 12B). The electrochemical ATRA of polychloroalkanes to olefins was achieved through a strategy demonstrated by Zeng [66] (Figure 12C), which was based on the synergistic combination of paired electrolysis and halogen bonding activation.

#### 2.1.6. Other Bonds Formation

**Figure 13:**
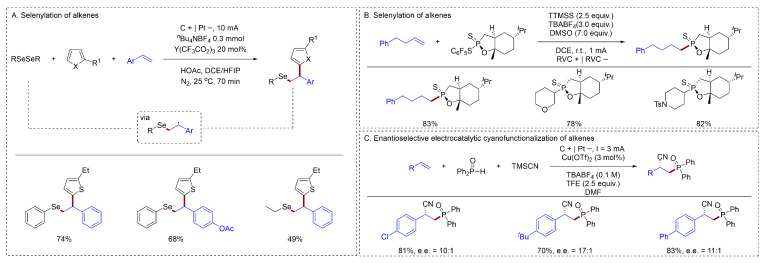
(**A**). Selenylation of alkenes; (**B**). Selenylation of alkenes; (**C**). Enantioselective electrocatalytic cyanofunctionalization of alkenes.

The synthesis of biologically active organoselenium species from alkenes has been reported. Lei reported a novel electrochemical radical selenylation of alkenes and activated arenes without external oxidants [67] (Figure 13A). The electrochemical activation of the Se–Se bond allowed for the complete conversion of the diselenide into selenium-centered radicals, facilitating a three-component radical carbo-selenation process. Baran reported an electrochemical protocol for alkene hydrophosphonylation [68] (Figure 13B). This study presented a novel strategy that forges the P(V) oxidation state directly, departing from conventional methods that rely on P(III) reagents. The transformations exhibited a broad scope due to their high chemoselectivity. Mechanistic insights revealed a bifurcated reactivity pathway, enabling selective access to either product from polyfunctional substrates. Lin reported the development of a highly enantioselective electrochemical cyanophosphinoylation of vinylarenes [49] (Figure 13C). Key to this advance was the identification of a new family of serine-derived chiral bisoxazolines bearing ancillary coordination sites, which rendered optimal ligand performance.

### 2.2. Cathodic Alkene Functionalization

#### 2.2.1. Reduction in Alkyl Halides

**Figure 14:**
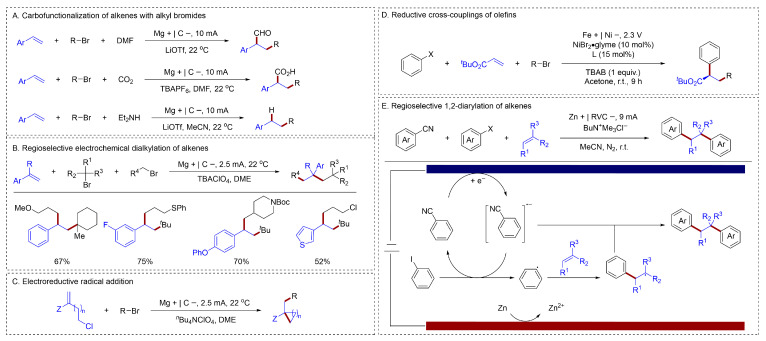
(**A**). Carbofunctionalization of alkenes with alkyl bromides; (**B**). Regioselective electrochemical dialkylation of alkenes; (**C**). Electroreductive radical addition; (**D**). Reductive cross-couplings of olefins; (**E**). Regioselective 1,2-diarylation of alkenes.

Cathodic reduction, as another means of electrochemical synthesis, has attracted increasing attention from chemists in recent years. Owing to their structural diversity and well-established synthetic routes, alkyl halides serve as versatile tools in organic synthesis and are particularly valued as excellent precursors for alkyl radicals. In 2020, Lin utilized an electroreductive strategy to accomplish the carbofunctionalization of radical-acceptor alkenes utilizing alkyl bromides [69] (Figure 14A). They achieved a radical-polar crossover pathway that enables the chemo- and regioselective addition of two distinct electrophiles across an alkene. These transformations employed readily accessible starting materials, such as alkyl halides and alkenes, under simple, transition-metal-free conditions, while exhibiting broad substrate scope and good functional group tolerance. This approach thus offered a new avenue for constructing Csp^3^–Csp^3^ bonds. Furthermore, Lin capitalized on the distinct electronic and steric properties of alkyl halides with different substitution patterns [70] (Figure 14B). They leveraged these differences to achieve selective activation and subsequent addition to alkenes under an electrochemical regime. Lin also reported a modular electrochemical approach for assembling a wide range of cyclic structures from simple alkene and alkyl halide precursors [71] (Figure 14C). This mild, general protocol proceeded through a radical-polar crossover pathway, enabling access to monocycles, benzo-fused rings, and spirocycles.

The asymmetric reductive difunctionalization of alkenes with alkyl bromides had also been developed. Mei reported a nickel-catalyzed, enantioselective electroreductive cross-coupling between acrylates and (hetero)aryl or alkyl halides [72] (Figure 14D). This method provided direct access to chiral α-aryl carbonyl compounds in high enantioselectivity and was notably effective with challenging aryl chlorides, a benchmark transformation that remained difficult by alternative approaches. Mechanistic studies, including cyclic voltammetry and electrode potential analysis, indicated that a Ni^I^ species activated aryl halides via oxidative addition and alkyl bromides through single-electron transfer.

In addition to direct reduction, the difunctionalization of alkenes and alkyl halides via indirect electroreduction has also been documented. Lei demonstrated a redox-mediated electrolysis that utilizes aryl nitriles as both aryl radical precursors and mediators [73] (Figure 14E). This strategy enabled a transition-metal-free, intermolecular 1,2-diarylation of alkenes with remarkable regioselectivity, activating a broad range of aryl halides (I, Br, Cl) to afford bibenzyl derivatives in high yields (up to 83%) and selectivity (>20:1 rr), as demonstrated by over 80 examples. The reaction commenced with the single-electron reduction in dicyanobenzene at the cathode to generate its radical anion (DCA•−). Subsequently, DCA•− transferred an electron to iodobenzene, yielding the corresponding aryl radical. This aryl radical then adds to the alkene in an anti-Markovnikov fashion, producing a benzyl radical intermediate. Next, this benzyl radical engaged in a radical/radical anion cross-coupling with another molecule of DCA•−. This coupling event afforded the final product and released a cyanide anion. Concurrently, at the anode, sacrificial zinc metal was oxidized to Zn^2+^.

#### 2.2.2. Hydrogenation of Alkenes

**Figure 15:**
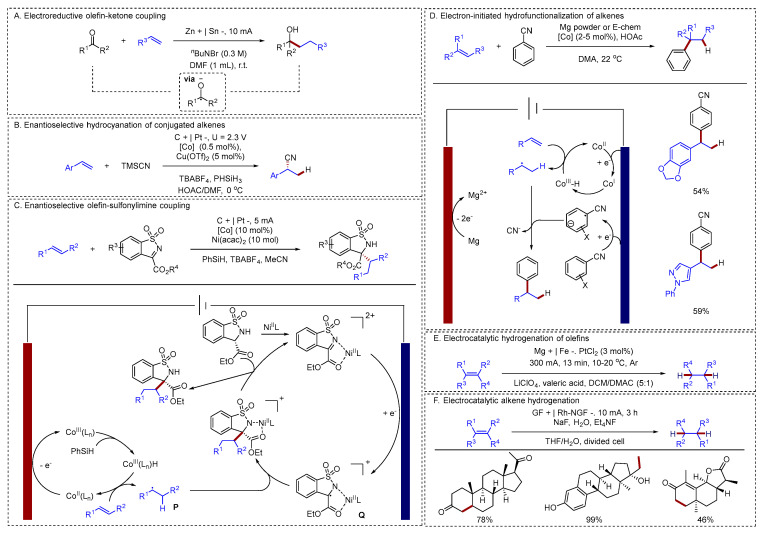
(**A**). Electroreductive olefin-ketone coupling; (**B**). Enantioselective hydrocyanation of conjugated alkenes; (**C**). Enantioselective olefin-sulfonylimine coupling; (**D**). Electron-initiated hydrofunctionalization of alkenes; (**E**). Electrocatalytic hydrogenation of olefins; (**F**). Electrocatalytic alkene hydrogenation.

Selective hydrogenation of alkenes plays a pivotal role in modern synthetic chemistry, enabling the production of sophisticated molecules used in pharmaceuticals and agrochemicals. In recent years, electrochemical hydrofunctionalization of alkenes has attracted considerable interest among chemists. Baran reported an electrochemical method for the hydrofunctionalization of alkenes and ketones [74] (Figure 15A). This work demonstrated that a ketone could serve as a nucleophile in addition to simple, unactivated olefins to achieve the same overall transformation. The electrochemical strategy enabled a broad coupling scope, and the reaction was notable for its scalability, chemoselectivity, and tolerance to both air and water. Alkene hydrocyanation had also been reported. Lin developed an asymmetric hydrocyanation of alkenes enabled by the combination of electrochemistry and cobalt catalysis [75] (Figure 15B). The electrocatalytic hydrocyanation reported by Lin represented a significant advance, highlighting electrochemistry’s unique role in uncovering new reactivities. This transformation would empower chemists by providing a powerful new tool for synthesizing diverse, enantioenriched nitriles. Similarly, Lu achieved the asymmetric hydrofunctionalization of alkenes by leveraging a combined electrochemical, cobalt, and nickel catalytic system [76] (Figure 15C). In this protocol, Co-catalyzed HAT from the anode was seamlessly merged with Ni-catalyzed sulfonylimine reduction at the cathode through efficient cross-coupling. The proposed mechanism began at the anode, where the catalyst precursor Co(II) was preferentially oxidized to a Co(III) species. This Co(III) intermediate then reacted with phenylsilane to form a Co(III)-H complex. Subsequently, the Co(III)-H species underwent metal-hydride hydrogen atom transfer (MHAT) with the olefin, generating carbon radical **P** and regenerating the Co(II) catalyst. Simultaneously at the cathode, the sulfonylimine—activated by coordination to a chiral Lewis acid—was reduced to form a radical anion. Finally, the enantioselective cross-coupling of radicals **P** and **Q** delivered the desired chiral amine product, accomplishing an asymmetric olefin–sulfonylimine coupling.

The mediation of hydrogen-atom transfer by earth-abundant transition-metal hydrides (M-Hs) has become a powerful methodology in organic synthesis. Lin reported a reductive method to generate Co-H [77] (Figure 15D). This strategy succeeded in intercepting the typical hydrogen evolution pathway, diverting the hydride to instead add across alkenes. These mechanistic insights, gleaned from electroanalytical and spectroscopic studies, directly enabled the invention of two novel alkene hydrofunctionalization reactions. In addition, electrochemical hydrogenation of alkenes had also been reported. Lu had developed an efficient Pt electrocatalyst from commercial PtCl_2_ that enabled the electrohydrogenation of olefins with protons and electrons at high current density [78] (Figure 15E). This approach offered exceptional functional group compatibility, requires minimal catalyst loading, and ensures precise, chemoselective synthesis of a wide range of valuable molecules. Lei had developed reusable, metal-modified GF-based electrodes for electrocatalytic alkene hydrogenation and deuteration, employing inexpensive and easily handled H_2_O/D_2_O as the hydrogen/deuterium source [79] (Figure 15F). These electrodes facilitated the reduction in mono- to tetra-substituted alkenes, exhibiting excellent chemoselectivity by tolerating various reducible functional groups (including Cl, Br, I, aldehyde, ketone, and cyano) during the C=C bond reduction.

## 3. Conclusions and Future Directions

Over the past decade, electrochemistry has made significant contributions as an important means to achieve high-value transformations of inexpensive olefins. The impressive advances in alkene functionalization are highlighted in a variety of recently published review articles. Anodic oxidation and cathodic reduction are two important modalities for achieving electrochemical alkene functionalization. Both approaches enable the high-value transformation of alkenes in an efficient and highly selective manner. This review summarizes recent advances in the electrochemical functionalization of alkenes, with a focus on reaction development and mechanistic insights.

While the field has seen impressive advances, key challenges must be addressed to unlock future opportunities:

(1) Current strategies predominantly rely on SET-enabled cleavage of heteroatom-heteroatom or carbon-halogen bonds to generate radicals, a process that demands relatively low energy compared to cleaving inert C–H or C–C bonds. While this facilitates efficient radical trapping by alkenes, it also presents an opportunity to advance classical radical chemistry by developing new reactions that involve the direct activation of C–H or C–C bonds to generate carbon-centered radicals for alkene difunctionalization.
(2) Most methodologies are largely applicable only to arylalkenes. There is a pressing need to develop general protocols that encompass a broader range of alkenes, particularly unactivated alkylalkenes and other unsaturated hydrocarbons like alkynes and enynes.
(3) The scope of terminating reagents is currently limited, which opens avenues for introducing novel functional groups as terminators to construct unprecedented molecular architectures.
(4) Selectivity Control: Advanced strategies for regioselective and enantioselective control are urgently needed, making the development of efficient chiral catalytic systems a top priority for achieving asymmetric synthesis.
(5) Industrialization remains a major challenge: the challenges lie in the design of reaction devices and the management of thermal effects in batteries, which are two key scientific issues to be addressed.

Looking ahead, we anticipate that synergistic strategies combining electrochemistry with transition-metal and/or photoredox catalysis (via SET, EnT, HAT) will drive the development of new cooperative electrocatalytic platforms. This integration is likely to overcome current limitations, significantly expanding the scope of substrates, initiating reagents, and terminating reagents. This review aims to provide critical insights into this dynamic field, and we expect a surge of innovative achievements in alkene difunctionalization in the coming years.

## Data Availability

No new data were created or analyzed in this study.
